# Potential Use of Amniotic Membrane - Derived Scaffold for Cerebrospinal Fluid Applications

**DOI:** 10.22088/IJMCM.BUMS.7.2.91

**Published:** 2018-08-19

**Authors:** Fereshteh Dorazehi, Mohammad Nabiuni, Hanieh Jalali

**Affiliations:** 1 *Department of Cell and Molecular Sciences, Faculty of Biological Sciences, Kharazmi University, Tehran, Iran.*; 2 *Department of Animal Biology, Faculty of Biological Sciences, Kharazmi University, Tehran, Iran* *.*

**Keywords:** Cerebrospinal Fluid, bone marrow mesenchymal stem cells, neural differentiation, amnion membrane derived scaffold

## Abstract

Scaffolds derived from decellularized tissues provide a natural microenvironment for cell culture. Embryonic cerebrospinal fluid (e-CSF) contains factors which play vital roles in the development of the nervous system. This research was aimed to survey the effect of Wistar rat e-CSF on neural differentiation of bone marrow derived mesenchymal stem cells (BM-MSCs) cultured on the human amniotic membrane (AM). BM-MSCs were collected from femurs and tibias, and were cultured in Dulbecco's Modified Eagle's Medium. The placenta was harvested from healthy women during cesarean section and AM was acellularized using EDTA and physical scrubbing. e- CSF was harvested from rat fetuses at E17. Adequate numbers of BM-MSCs were cultured on acellularized membrane, and were treated with E17 CSF for 7 days. MTT (3-(4, 5-dimethylthiazol-2-yl)-2.5-diphenyltetrazolium bromide) assay confirmed the survival and proliferation of BM-MSCs cultured on AM derived scaffold. Hematoxylin/eosin staining and scanning electron microscopy showed the morphological and the structural changes of BM-MSCs throughout the culture and treatment with e-CSF. The results of immunocytochemistry showed that microtubule associated protein 2 and beta-III tubulin were expressed in BM-MSCs cultured on acellular amnion scaffold and treated with e-CSF. Our results showed for the first time that the combination of acellular AM as a natural scaffold and e-CSF as a source of neurological factors could effectively improve the BM-MSCs cultivation and differentiation

The extracellular matrix-based scaffolds act as an excellent three dimensional media forstem cell cultures (1, 2). Scaffolds derived from decellularized tissues are a viable alternativeto synthetic polymers, and provide a natural microenvironment for cell migration and differentiation (3, 4). Amniotic membrane (AM) is a thin, elastic, semi-transparent, and semi-permeable fetal derived tissue which is attached to the chorion (5). AM is an easy harvesting tissue and has significant properties such as anti-inflammatory, immunomodulatory, and antimicrobial effects, and is considered as a fascinating biomaterial in the field of tissue engineering and regenerative medicine (6). The extracellular matrix of AM contains collagen type IV, type VII, fibronectin, and laminins-1 and -5, and provide a natural scaffold for cell adhesion and proliferation (7).

Cerebrospinal fluid (CSF) bulk of the central nervous system (CNS) comprises the most extracellular fluid of the brain, and fills brain ventricles, subarachnoid space, and the spinal canal (8). CSF is important for the physical protection of a developing brain, and CSF sufficient pressure maintenance in the ventricle is also essential for normal CNS development (9). In addition, CSF circulation applies ''trophic'' effect on the developing brain by providing important growth factors and other biological active substances (10). In adults, CSF has been considered as an intermediary between blood and brain for transmitting the nutrients and growth factors, and as a liquid buffer to protect the brain and also major vessels that carry the blood to the brain. It was reported that CSF contains nerve growth factor (NGF) and transforming growth factor α (TGF-α) (11). As a whole, the available data indicates the presence of diffusible factors in embryonic CSF (e-CSF) to regulate survival, proliferation, and differentiation of neuroepithelial stem cells (12-14). Some studies introduced e-CSF as a transporter for the important cytokines as NGF, TGFβ, brain derived neurotrophic factor (BDNF), neurotrophin-3 (NT-3), and insulin-like growth factor (IGF) while these factors play a vital role in cell proliferation, migration, differentiation, and development of the nervous system (15). Zhu et al. reported a simple and natural way in which human CSF can increase the proliferation, migration, and viability of exogenous primary human adipose-derived mesenchymal stem cells (hAMSCs) and human fetal-derived neural progenitor cells (hfNPCs) (16). The results of their research provide insight into developing the clinical efficacy of CSF for the treatment of CNS pathologies.

Mesenchymal stem cells (MSCs) are adult multipotent stem cells that were demonstrated to be present in a variety of tissues such as bone marrow (17-19), umbilical cord blood (20), amniotic membrane and fluid (21), and adipose tissue (22). Bone marrow MSC (BM-MSCs) are best characterized and well-established source of MSCs (23); they express and differentiate into three lineage related cells in culture media (24).

Using an *in vitro* three-dimensional model, we aimed to explore the effect of e-CSF as a rich medium of neural growth factors on the fate of BM-MSCs, and investigate the role of extracellular matrix of AM as a natural scaffold on the proliferation and differentiation of BM-MSCs treated with e-CSF.

## Materials and methods

**CSF collection**

CSF was collected from Wistar rat embryos at E17. E17 was selected for CSF collection according to previous results showing that total protein concentration of E17 CSF was significantly greater than other embryonic days, and had most significant effect on cell proliferation, viability, and differentiation of BM-MSCs (25). CSF was collected from the cisterna magna using glass micropipette (Wheaton, USA), and to remove the remaining cells and debris, samples were centrifuged at 1500 rpm (Hettich, Germany). The supernatants were transferred into sterile microtubes (Sorfa, China) and were immediately frozen at - 86°C (Smart Tech, Canada). To prevent protein degeneration, all stages were carried out on ice. All procedures were carried out according to the guidelines of National Research Council of Iran, 2013-91126/48902. The study was approved by  the Ethics Committee of Kharazmi University (9446/25.12.1393).

**Isolation and expansion of BM-MSCs**

BM-MSCs were obtained from femurs and tibias of 4 to 6 weeks old Wistar rats. Muscles and tissues around the bones have been removed by scalpel (Mehrazmalab, Iran), and stromal cell suspension of bone marrow was prepared by flushing the femurs and tibias using a syringe and 22-gauge needle into Dulbecco Modified Eagle Medium (DMEM) (Sigma-Aldrich, United Kingdom) supplemented with 15% fetal bovine serum (FBS) (Gibco, Life Technologies, Paisley, United Kingdom), 50 U/mL penicillin, and 50 mg/mL streptomycin (Gibco BRL, Life Technologies, Paisley, United Kingdom). The suspension was cultivated in 25 cm^2 ^flasks (Sorfa, China), and incubated at 37 °C in 5% CO_2 _(Binder, Germany). After 24 h, media were replaced with fresh medium, and the non-adherent cells were removed using extensive washing by phosphate buffer saline (PBS) (Gibco- Invitrogen, United Kingdom). Harvested BM-MSCs were characterized, and their mesenchymal identity was confirmed according to our previous study. Briefly, BM-MSCs were detected by flow cytometry analysis of specific surface antigens of cluster of differentiation (CD) CD45, CD44, and CD29. However, their differentiation potential into adipocytes and osteocytes was conducted by culturing in culture media supplemented by differentiation inducing factors (25).

**Scaffold preparation**

Human placenta was obtained from healthy donors during caesarian sections under sterile conditions, and placed immediately in sterile normal saline (Samen, Iran) containing antibiotics. The AM was separated from other associated membranes of the placenta in a class 2 safety cabinet (Microflow, United Kingdom). Multi-stage washing was performed with normal saline containing penicillin and streptomycin until tissue clearing. It was cut approximately into 3 × 3 cm pieces, and was placed from the stroma on cellulose filter paper (Whatman, England) with the epithelium facing-up, then was maintained in a vial containing equal ratios of DMEM /glycerol (Thermo Fisher Scientific, USA), and stored at -80 °C (6). To prepare the scaffold, amniotic membrane AM was decellularized with the following procedure. Frozen AM samples were thawed at 37 °C, washed with PBS, and then incubated in 0.25% trypsin- EDTA at 37 °C for 20 min. For complete removing of cells, the membrane was gently scraped with cell scraper (SPL, Korea). The acellular AMwas transferred into DMEM, and incubated for 24 h at 37 °C in 5% CO_2_.

**Implantation of BM-MSCs on AM scaffold**

The decellularized AM pieces were spread on culture plates, and 2×10^5^ cells of BM-MSCs were transferred and cultured on them in DMEM containing 1% penicillin/streptomycin (Gibco-invitrogen, United Kingdom) and 15% FBS for 30 days. Monitoring under invert microscope (Ziess, Germany) was done to make sure of cell adhesion to the scaffold. Nonadherent cells were removed through washing by medium.

**Hematoxylin and eosin staining of AM**

After three times washing with PBS, scaffolds were fixed for 1-2 h in Bowen buffer (Sigma-Aldrich, United Kingdom). The samples were dehydrated with ascending grades of alcohol (Jahan alcohol Teb, Iran) (25%, 50%, 75%, 85%, 95%, and 100%), then stained with hematoxylin and eosin (Merck, Germany). Specimens were rinsed with alcohol, 90% and 100%, respectively and finally, were photographed by an optical microscope (Nikon, Japan).

**MTT proliferation assay**

To investigate the proliferation rate of BM-MSCs seeded on AM, the 3-(4, 5-dimethylthiazol-2-yl)-2.5-diphenyltetrazolium bromide (MTT; Sigma-Aldrich, United Kingdom) colorimetric assay was performed. Briefly, 2×10^5^ cells were seeded on AM derived scaffold, and after allowing for cell adherence and proliferation, MTT assay was carried out at fifth, seventh, tenth, fifteenth and thirtieth days after cell implanting. A total volume of 20 μL of 5 mg/mL MTT solution was added to each well and incubated for 4 h, and then the medium was discarded. Subsequently, 150 μL dimethyl sulfoxide (DMSO; Merck-Germany) was added to dissolve formazan salts, and the absorbance value for each well was measured at 490 nm (UNICO, USA). The cell survival on the scaffold was calculated from the following formula:

Viability= mean absorbance of sample /mean absorbance *100

**Treatment with e-CSF**

To study the effect of e-CSF on BM-MSCs seeded on AM derived scaffold, 5 days after implantation, cells were treated with a concentration of 7% v/v of E17 CSF as test group or with basic fibroblast growth factor (b-FGF) (10 ng/ml) (Sigma-Aldrich, United Kingdom) as control group. In this way, the supernatant was removed and replaced with 1 ml fresh medium containing e-CSF or b-FGF. On the seventh day of treatment, cells were examined for neuronal differentiation.

**Scanning electron microscopy**

The scaffolds were fixed with 2.5 M glutaraldehyde (Sigma-Aldrich, United Kingdom) in 0.1% PBS at 4 ^°^C for 1 h and then post-fixed in 1% osmium tetroxide (Merck, Germany) for 2 h. Following fixation, specimens were dehydrated in ascending grades of ethanol (25%, 50%, 75%, 85%, 95%, 100%), critical point dried, sputtered with gold and viewed with a TESCAN scanning electron microscope.

**Immunocytochemistry**

The expression of neural related proteins microtubule associated protein 2 (MAP-2) and beta-III tubulin in e-CSF treated BM-MSCs cultured on AM scaffolds were analyzed using immunocytochemistry. Briefly, cells cultured on scaffolds were fixed in 4% paraformaldehyde (Merck, Germany) in PBS for 45 min, permeabilized with 0.4% Triton X-100 (Merck, Germany) for 30 min at room temperature, and subsequently blocked with 1% bovine serum albumin (BSA; Sigma-Aldrich, United Kingdom) in TPBS [Tween 20 (Sigma-Aldrich, United Kingdom) in PBS] for 1 h at room temperature. Specimens were incubated at 4 °C overnight in the presence of either anti-MAP-2 (1:500 dilution; Sigma- Aldrich, Poole, UK) or anti-beta III tubulin mAb (1:500 dilution; Sigma- Aldrich, Poole, UK) monoclonal antibodies. The following day, after three washings with TPBS, fluorescein isothiocyanate (FITC) conjugated goat anti-mouse secondary antibody (1:1000 dilution; Sigma- Aldrich, Poole, UK) was added at room temperature for 1 h. Scaffolds were then washed and photomicrographs were taken with a fluorescence microscope (Olympus, Tokyo, Japan). The light intensity of the fluorescent immunocytochemistry images was measured using the Image J software.

**Statistical analysis**

Quantitative statistical analysis was performed using one-way ANOVA and TUKEY test and significance was accepted for P values of <0.05.

## Results

**BM-MSCs harvesting and culturing**

**Fig. 1:**
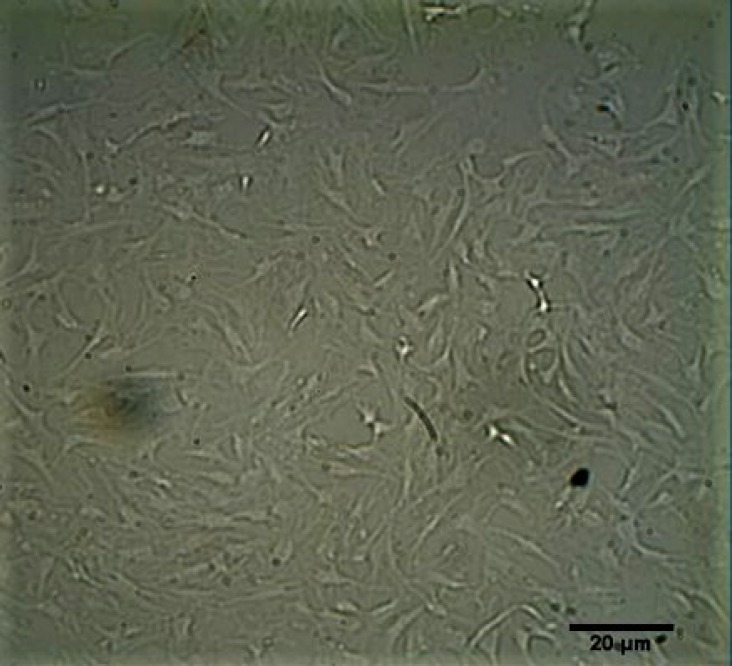
Morphological characteristics of BM-MSCs. BM-MSCs on the first passage are slender and fibroblast-like (×100).

Bone marrow mesenchymal stem cells were harvested from femur bone of Wistar rat and transferred into culture plates which contained spherical-shaped objects and aggregated cells. After the second passage, debris and non-adherent cells were removed and a homogenous population of BM-MSCs was observed (Fig. 1).

**AM scaffold characterization**

**Fig. 2:**
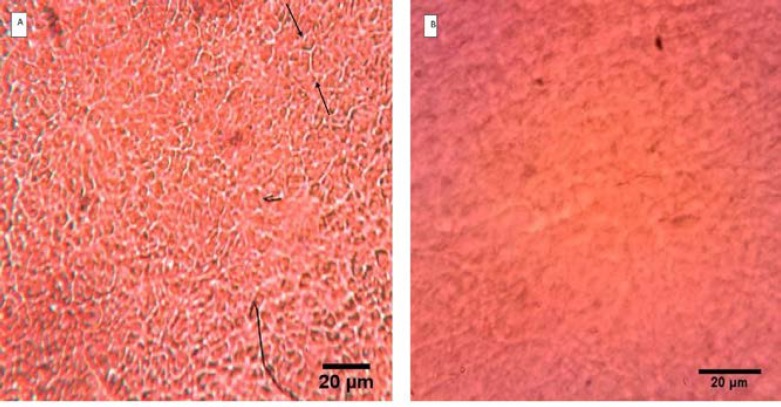
Hematoxylin-eosin staining of human amniotic membrane. A: intact amniotic membrane covered with a layer of epithelial cells which are marked with black arrows; B: dAM without epithelial cells (40 ×)

**Fig. 3:**
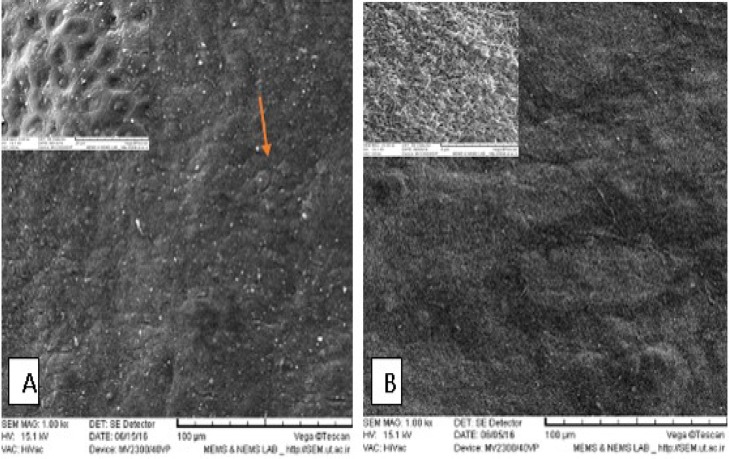
Electron microscopy photomicrograph of human amniotic membrane. A: intact human amniotic membrane (AM); arrow shows the epithelial cells of AM; B: decellularized AM without surface epithelial layer

To confirm complete removing of epithelial cells, hematoxylin- eosin staining was done. Observations revealed that there were no cell fragments or epithelial cells in decellularized AM (dAM) (Fig. 2). Scanning electron microscopy images confirmed the process of decellularization and dAM had a porous non cellular structure (Fig. 3).

**Fig. 4:**
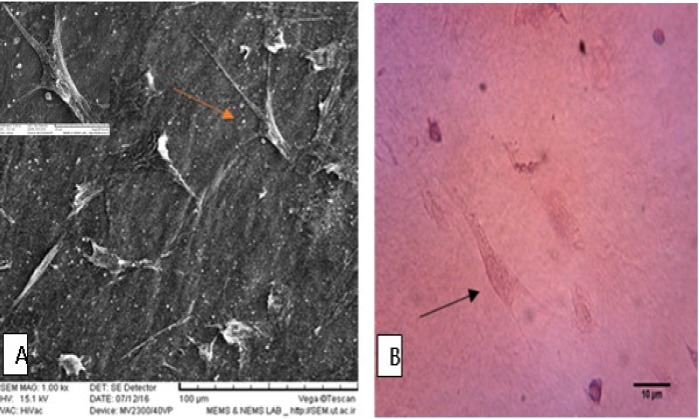
BM-MSCs cultured on decellularized AM. A: scanning electron microscopy micrograph; B: hematoxylin and eosin staining, Arrows show BM-MSCs-seeded on AM scaffold

**Fig. 5:**
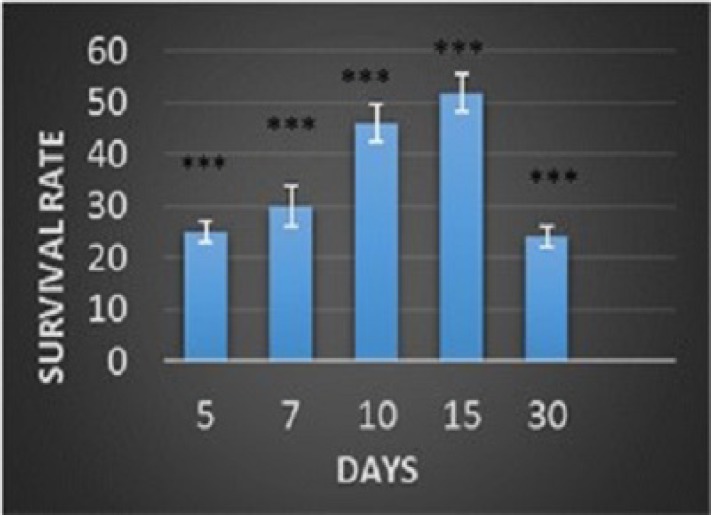
The proliferation rate of BM-MSCs after seeding on amniotic membrane scaffold. The proliferation of BM-MSCs on the scaffold was significantly high on the 5th, 7th, 10th, and 15th days compared with the first day but it dropped on the 30th day of implantation (P <0.001). Data are shown as mean ± SD

**Fig. 6:**
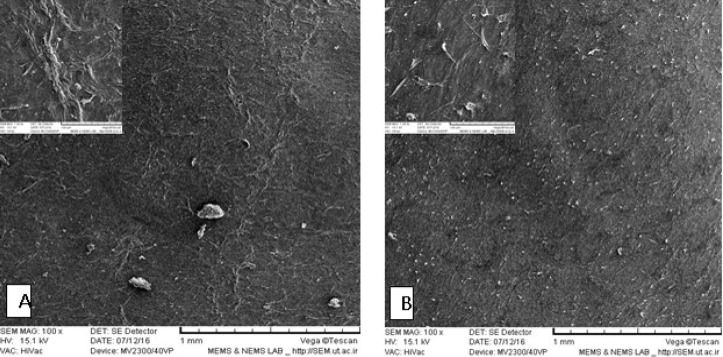
Scanning electron microscopy photograph of BM-MSCs seeded on amnion membrane. A: medium containing e-CSF; B: normal medium

**Fig. 7:**
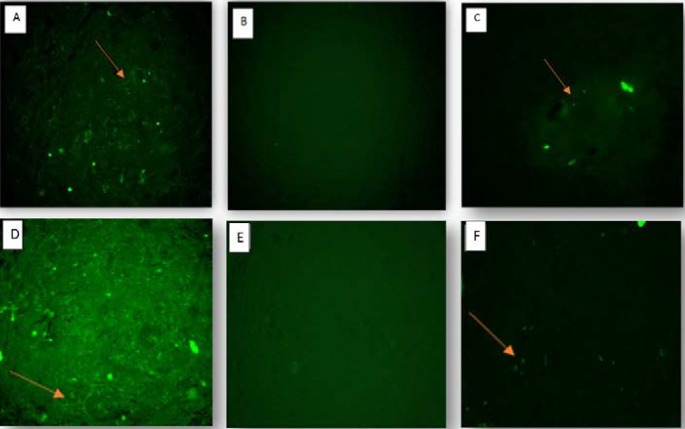
The expression of MAP-2 and beta-III tubulin in BM-MSCs cultured on amniotic membrane scaffold under treatment with E17 CSF. A: MAP-2 in e-CSF treated cells; B: MAP-2 in non-treated cells; C: MAP-2 in bFGF treated cells; D: beta-III tubulin in e-CSF treated cells; E: beta-III tubulin in non- treated cells; FF: beta-III tubulin in bFGF treated cells (100 ×)

**Fig. 8:**
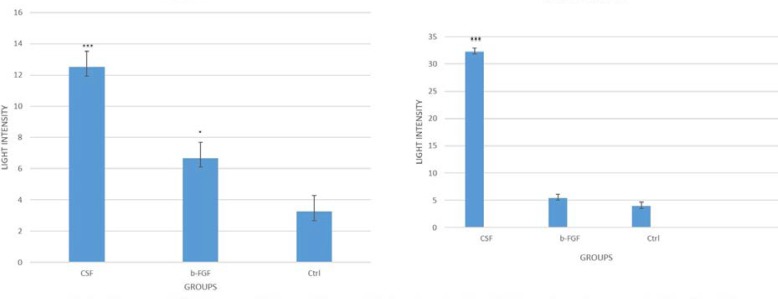
Comparison of MAP-2 and beta-III tubulin expression in BM-MSCs treated with e-CSF and bFGF using fluorescent light microscopy. (* P < 0.05; *** P < 0.001)

**Seeding of BM-MSCs on dAM**

Twenty- four hours after AM preparation, BM- MSCs were seeded on dAM. Scanning electron microscopy images detected the fibroblast like BM-MSCs attached to dAM (Fig. 4A). Furthermore, hematoxylin-eosin staining showed the presence of spindle-shaped BM-MSCs on AM scaffold in culture medium (Fig. 4B).

**Proliferation of BM-MSCs on dAM**

Evaluation of survival and proliferation rate of BM-MSCs cultured on AMderived scaffold revealed that the viability of BM-MSCs increased significantly throughout 15 days. However, the rate of proliferation decreased during the next 15 days of culture, and was significantly higher in comparison with the first day of culture (P <0.001) (Fig. 5).

**Neural differentiation of BM-MSCs treated with e-CSF in three dimensional medium**

The BM-MSCs were cultured on AM derived scaffold in the presence of e-CSF for 7 days and their neural differentiation was evaluated with scanning electron microscopy and immunocytochemistry methods. Scanning electron microscopy images showed a neuron-like morphology of BM-MSCs, and presence of the cell processes which were comparable with non-treated cells (Fig. 6). Immunofluorescence staining showed that BM-MSCs differentiated into neurons in the presence of e-CSF, expressed two neural related markers. After exposure to neurogenic medium, the percentage of the cells expressing MAP-2 and beta-III tubulin was significantly greater in the e-CSF containing culture condition than in the b-FGF containing medium. No expression of neural markers was observed in normal three dimensional culture of BM-MSCs (Fig. 7). The Image J. analysis of fluorescent density revealed that e-CSF promoted the expression of MAP-2 and beta-III tubulin significantly more than b-FGF (Fig. 8).

## Discussion

Several studies reported the presence of neurogenic and neurotrophic factors in the CSF, which take part in nervous system development, proliferation, migration, and differentiation of nerve cells (26, 27). It was also shown that e-CSF protein structure is much more complex than mature CSF, which include growth factors and cytokines that are effective in cell behavior regulation. One of this neurogenic factors is NGF that has been shown to be present in chick e-CSF, and its level was reported to increase in 17 and 18 embryonic days (28, 29). Laboratory findings showed that change and, more importantly, loss of e-CSF strongly reduce nerve cells populations in the nervous system (9). In general, CSF has a key role as a liquid path for soluble signals transmission, and in this manner, it affects brain parenchyma cells behavior (30). Moreover, it has been demonstrated that e-CSF as a neural stem cell niche is capable to provide a microenvironment regulating neuroepithelial cells, and supporting viability, proliferation and differentiation of primary rat cortical cells (31), PC12 cells (32), and BM-MSCs (25).

The significant and most valuable outcome in the current study was that the BM-MSCs-seeded onto a natural scaffold treated by e-CSF, developed morphological changes into neuron-like cells by a week, and our light intensity analysis showed that the expression of MAP-2 and beta-III tubulin neural markers significantly increased in comparison with both bFGF treated and non-treated cells. Although our results were in accordance with previous studies showing the role of CSF in neural differentiation of stem cells (33, 34), we used e-CSF for the first time in a three-dimensional culture medium to induce neural differentiation in BM-MSCs.

The neural differentiation of BM-MSCs has been analyzed by different researchers, either in conventional two-dimensional (2D) culture (35, 36) or three-dimensional (3D) culture (37-39). In the current study, AM-derived scaffold was used to 3D culture of BM-MSCs. For AM decellularization, and blocking the activity of proteases during cell lysis, EDTA containing trypsin was used in our procedure. In this decellularization process, collagen, elastin, and glycosaminoglycans are not damaged (40,41), and the interconnected pore networks within the scaffold are kept to provide the spatial position of the interactions of cytokines and growth factors released from cells (42,43).

According to the scanning electron microscope, acellular amniotic scaffold pore size ranges between 44-160 μm, and the average pore size is 90 μm and shows the average porosity of 90 percent. Regarding the size of mesenchymal stem cells that varies between 20-40 μm, the scaffold can be appropriate for cell infiltration, gas, and food exchange (43). Our scanning electron microscopy experiments showed that acellular AM had preserved its porous network of collagen, and spindle-shaped BM-MSCs tended to penetrate into its matrix to establish cell-cell and cell-matrix connections. Also, AM has the ability to provide a suitable environment to support proliferation and survival of BM-MSCs, so the proliferation and survival of the cells continued until the 15^th^ day (unpublished data), and the cell viability declined on the 30^th^ day. The evidence from previous studies indicates that scaffolds may show toxicity and prevent cell proliferation (44), however, our findings detected no evidence of toxicity, and cell viability lasted for 15 days. Occasionally, when the culture time prolonged, cell viability in 3D cultures declined slightly. This may be due to changes in the structure of scaffold that may result in the the lack of oxygen and nutrients as well as the accumulation of waste in the scaffold (45).

In conclusion, our results provided evidence that the combination of acellular AM, as a natural scaffold, and e-CSF, as a source of neurological factors, could effectively improve the BM-MSCs cultivation and neural differentiation. Our findings could be useful in improving research and/or clinical applications of CSF in the context of the neural differentiation of stem cells.
